# Changing Frequency of the Use of Intraoperative Equipment Among Neurosurgeons, Neurosurgery Residency Faculty, and Neurosurgery Residents

**DOI:** 10.7759/cureus.25406

**Published:** 2022-05-27

**Authors:** Paras Savla, Tye Patchana, Andrew Ku, James Brazdzionis, James Wiginton, Dan E Miulli

**Affiliations:** 1 Neurosurgery, Riverside University Health System Medical Center, Moreno Valley, USA; 2 Neurosurgery, California University of Science and Medicine, Colton, USA; 3 Neurosurgery, Arrowhead Regional Medical Center, Colton, USA

**Keywords:** neurosurgery residency, neurosurgical residency, neurosurgical training, medical education, surgical loupes, loupes

## Abstract

Neurosurgical procedures have relied on the use of various intraoperative equipment since its advent. These include an operative microscope, ultrasound, and loupes with a headlight. The necessity of these pieces of equipment makes them vital in the training of residents as well. A national survey utilizing a Likert scale to determine how often loupes, microscopes, and ultrasound were used for various neurosurgeries was created. This was then compared to a single program’s responses, and it identified that the practice parameters of residents closely modeled those behaviors portrayed by their attending mentors. It appears that the higher frequency of use by residents when compared to faculty and neurosurgeons nationwide highlights the importance of this equipment in training neurosurgical residents.

As such, they should be available to residents from the onset of training to promote the highest quality of learning. Faculty should encourage the use of this equipment by leading by example, and residents, in turn, should use all the available equipment as often as possible to maximize the quality of their training. Modulating the use of learning technologies can be accomplished if it is a nationally accepted practice, discussed in an academic setting with the residents, and modeled by the faculty.

## Introduction

Neurosurgical procedures have relied on the use of various intraoperative equipment since its advent. The complexity of neurosurgical procedures with narrow operative corridors and the need for microscopic approaches necessitated the usage of technologies to improve visualization of the surgical field. Some of the baseline technologies for neurosurgical procedures include surgical loupes with or without a headlight, a surgical microscope, and ultrasound. Additionally, endoscopic and neuroendovascular adjunct technologies are also being used, albeit they are less of a staple in the operating room. The cost of all these devices, however, has been increasing. Typically, a hospital takes on the responsibility of purchasing a surgical microscope and ultrasound as these pieces of equipment reside in the hospital. Operative loupes are typically purchased by individual surgeons and assistants as the interpupillary distance, prescription, and operating distance are unique to everyone. Loupes come in a wide array of magnification and working distances, which enhances the detail of the working surgical field and offers improved ergonomics secondary to the working distance.

Unlike surgical microscopes, in many designs, these cannot be adjusted. Individualized loupes have been found to promote improved surgical ergonomics compared to fixed standard loupes, producing fewer work-related musculoskeletal disorders [1]. While loupes are not accounted for within hospital expenses (and therefore fall onto individuals), they still bring a great benefit to surgical services, particularly when operating on structures between 1 and 10 mm that require magnification but do not warrant the use of microscopes [2]. Residents often begin using loupes in training. We sought to quantify the use of loupes for both residents and attendings, both within training and practice. Further, we wished to qualify the practice parameters in usage by the residents of loupes and other technology within a single program and identify whether they appropriately modeled the behavior of their attending surgeons. We also sought to identify whether resident usage of these technologies modeled national trends.

## Materials and methods

A national survey utilizing a Likert scale to determine how often loupes, microscopes, and ultrasound were used for various neurosurgeries was created, utilizing Survey Monkey (a free online survey software, www.surveymonkey.com). This survey was used to assess the utilization of intraoperative ultrasound, microscope, and loupes (with and without headlight). Following several iterations by faculty members, the survey was sent to 102 Accreditation Council for Graduate Medical Education (ACGME) Neurosurgery programs in the United States. We received a total of 37 neurosurgery attending responses. We received a total of 12 responses from the faculty of our institution, the Riverside University Health System (RUHS) Neurosurgery department. Eleven neurosurgery residents responded to the survey, and nine residents responded to the post-survey, which was conducted one month later. The survey questions are depicted in Table 1. Statistical analysis was completed using Microsoft Excel, whereby simple percentages based on survey responses were calculated. These percentages were compared between groups to investigate the similarities in usage. A sample of the survey questions is presented in the appendix figure.

**Table 1:** Percentage of respondents who responded “Always” or “Frequently” to operations involving loupes, not including operations not performed

	Loupes with headlight	Cranial epidural hematoma	Cranial subdural hematoma	Brain abscess or tumor resection	Cortical dissection >1 cm but <3 cm	Intradural spinal cord tumor	Spinal epidural hematoma	Carpal tunnel or cubital tunnel surgery	Clipping of a circle of Willis aneurysm
Nationwide	64%	77%	74%	74%	68%	64%	71%	80%	57%
Faculty	67%	58%	50%	50%	50%	50%	58%	75%	29%
Resident	73%	100%	100%	100%	100%	100%	100%	100%	100%
Resident post-survey	100%	100%	100%	100%	100%	100%	100%	100%	100%

## Results

Ultrasound

**Figure 1:**
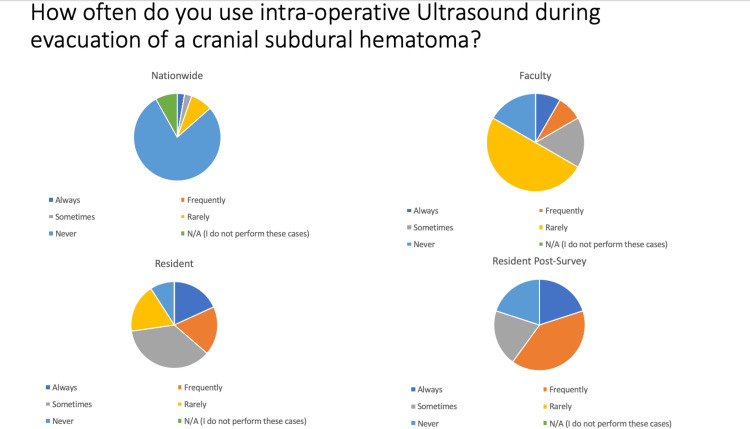
Pie charts demonstrating the use of intraoperative ultrasound for evacuation of subdural hematomas by attending neurosurgeons, faculty, and pre- and post-survey residents

In regard to the use of ultrasound for evacuation of cranial epidural hematomas, 6% (2/34) of attending surgeon respondents nationwide, 25% (3/12) of faculty, 64% (7/11) of pre-survey, and 78% (7/9) of residents post-survey said they “Always” or “Frequently” used ultrasound (US). In regard to cranial subdural hematomas, 3% (1/34) of attending surgeon respondents nationwide, 17% (2/12) of faculty, 36% (4/11) of pre-survey, and 60% (6/10) of residents post-survey used US (Figure 1). For brain abscess or resection of tumors, 26% (9/34) of attending surgeons nationwide, 92% (11/12) of faculty, 91% (10/11) of pre-survey, and 100% (10/10) of post-survey residents used US. For cortical dissection > 1 cm but <3 cm, 19% (6/32) of attendings nationwide, 83% (10/12) of faculty, 73% (8/11) of pre-survey, and 90% (9/10) of post-survey residents used US. For intradural spinal cord tumors, 47% (15/32) of attending surgeons nationwide, 92% (11/12) of faculty, 82% (9/11) of pre-survey, and 90% (9/10) of post-survey residents used US. For spinal epidural hematomas, 20% (6/30) of attending surgeons nationwide, 50% (6/12) of faculty, 55% (6/11) of pre-survey, and 80% (8/10) of post-survey residents used US. Lastly, for carpal/cubital tunnel surgery, the results were 0% (0/16) nationwide, 0% (0/7) for faculty, and 33% (2/6), then 50% (3/6) for residents.

Intraoperative microscope

**Figure 2:**
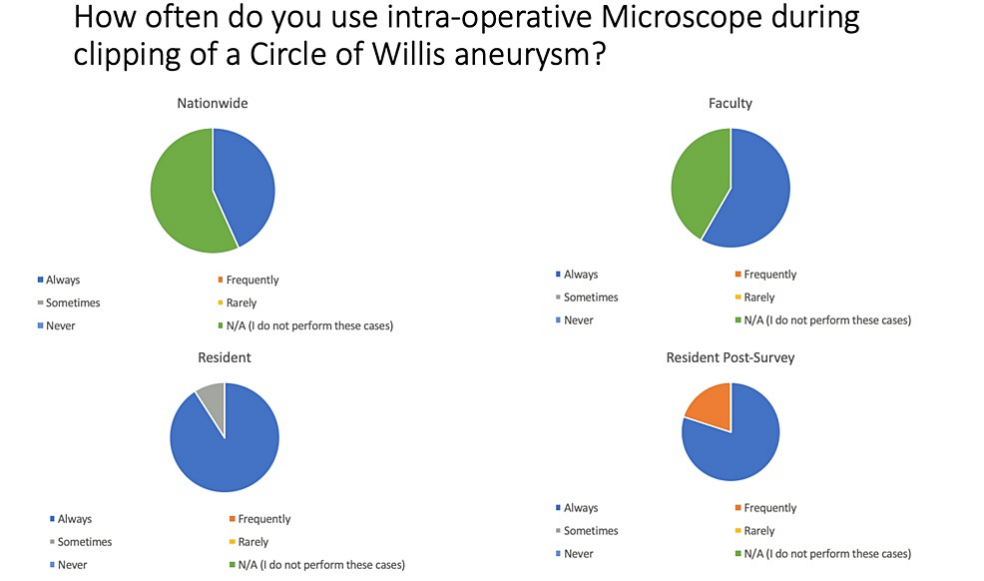
Pie graph depictions of usage of intraoperative microscopy during clipping of the circle of Willis aneurysms by attending neurosurgeons, faculty, and pre- and post-survey residents

Regarding the use of microscopes for clipping of circle of Willis aneurysms, 100% (16/16) of attending surgeon respondents nationwide, 100% (7/7) of faculty, 91% (10/11) of pre-survey, and 100% (10/10) of post-survey residents said they “Always” or “Frequently” used intraoperative microscopes (Figure 2). For cranial subdural hematomas, 3% (1/35) of attending surgeons nationwide, 0% (0/12) of faculty, 0% (0/11) of pre-survey, and 30% (3/10) of post-survey residents used intraoperative microscope. For either brain abscess or resection of tumor(s), 71% (24/34) of attending surgeon respondents nationwide, 92% (11/12) of faculty, 82% (9/11) of pre-survey, and 100% (10/10) of post-survey residents endorsed the use of intraoperative microscopy. For cortical dissection > 1 cm but <3 cm, 68% (23/34) of attending surgeons nationwide, 83% (10/12) of faculty, 100% (11/11) of pre-survey, and 100% (10/10) of post-survey residents endorsed the use of the microscope.

For intradural spinal cord tumors, 97% (32/33) of attending surgeon respondents nationwide, 92% (11/12) of faculty, 100% (11/11) of pre-survey, and 100% (10/10) of post-survey residents endorsed the use of intraoperative microscopy. For spinal epidural hematomas, 22% (7/32) of attending surgeons nationwide, 25% (3/12) of faculty, 55% (6/11) of pre-survey, and 60% (6/10) of post-survey residents endorsed the use of intraoperative microscopy. For spinal microdiscectomy, 96% (26/27) of attending surgeon respondents nationwide, 92% (11/12) of faculty, 91% (10/11) of pre-survey, and 90% (9/10) of post-survey residents endorsed the use of intraoperative microscopy. Finally, regarding carpal/cubital tunnel surgery, 7% (1/14) of attending surgeon respondents nationwide, 0% (0/8) of faculty, 25% (2/8) of pre-survey, and 43% (3/7) of post-survey residents endorsed the use of the intraoperative microscope.

Intraoperative loupes

**Figure 3:**
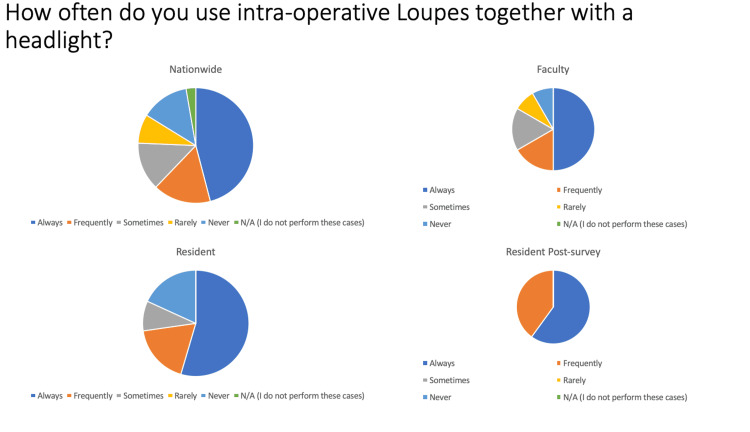
Pie graph depictions of the use of intraoperative loupes and headlights by attending neurosurgeons, faculty, and pre- and post-survey residents

In regard to the use of intraoperative loupeswith a headlight, 64% (23/36) of attending surgeon respondents nationwide, 67% (8/12) of faculty, 73% (8/11) of pre-survey, and 100% (10/10) of post-survey residents said they “Always” or “Frequently” used both headlights and loupes together (Figure 3). For cranial epidural hematomas, 77% (27/35) of attending surgeon respondents nationwide, 58% (7/12) of faculty, 100% (11/11) of pre-survey, and 100% (10/10) of post-survey residents endorsed the use of intraoperative loupes and headlight. In regard to cranial subdural hematomas, 74% (26/35) of attending surgeons nationwide, 50% (6/12) of faculty, 100% (11/11) of pre-survey, and 100% (10/10) of post-survey residents endorsed the use of loupes and headlight.

Regarding brain abscess or resection of tumors, 74% (26/35) of attending surgeons nationwide, 50% (6/12) of faculty, 100% (11/11) of pre-survey, and 100% (10/10) of post-survey residents endorsed the use of loupes and headlight. For cortical dissection > 1 cm but <3 cm, 68% (23/34) of attending surgeon respondents nationwide, 50% (6/12) of faculty, 100% (11/11) of pre-survey, and 100% (10/10) of post-survey residents endorsed the use of interoperative loupes and headlight. In regard to intradural spinal cord tumors, 64% (21/33) of attending surgeons nationwide, 50% (6/12) of faculty, 100% (11/11) of pre-survey, and 100% (10/10) of post-survey residents utilized loupes and headlight. In the evacuation of spinal epidural hematomas, 71% (22/31) of attending surgeons nationwide, 58% (7/12) of faculty, 100% (11/11) of pre-survey, and 100% (10/10) of post-survey residents endorsed loupes and headlight usage. In regard to carpal/cubital tunnel surgery, 80% (12/15) of attending surgeons nationwide, 75% (6/8) of faculty, 100% (9/9) of pre-survey, and 100% (9/9) of post-survey residents endorsed the use of intraoperative loupes and headlights. Lastly, for clipping of a circle of Willis aneurysm, 57% (8/14) of attending surgeons nationwide, 29% (2/7) of faculty, 100% (11/11) of pre-survey, and 100% (10/10) of post-survey residents endorsed the use of intraoperative loupes and headlights.

In regard to the evaluation of those not using loupes nor microscopes for evacuation of cranial epidural hematoma, 17% (6/35) of attending surgeons nationwide, 25% (3/12) of faculty, 10% (1/10) of pre-survey, and 30% (3/10) of post-survey residents stated they “Always” or “Frequently” used neither loupes nor microscopes for cranial epidural hematomas. For cranial subdural hematomas, 11% (4/35) of attending surgeon respondents nationwide, 17% (2/12) of faculty, 9% (1/11) of pre-survey, and 30% (3/10) of post-survey residents endorsed using neither loupes nor microscopes. For evacuation of brain abscess or resection of tumors, 6% (2/35) of attending surgeon respondents nationwide, 8% (1/12) of faculty, 9% (1/11) of pre-survey, and 30% (3/10) of post-survey residents endorsed using neither loupes nor microscopes. In regard to craniotomy for cortical dissection > 1 cm but <3 cm, 3% (1/34) of attending surgeon respondents nationwide, 8% (1/12) of faculty, 9% (1/11) of pre-survey, and 30% (3/10) of post-survey residents endorsed using neither loupes nor microscopes. For resection of intradural spinal cord tumors, 0% (0/34) of attending surgeon respondents nationwide, 8% (1/12) of faculty, 10% (1/10) of pre-survey, and 30% (3/10) of post-survey residents endorsed using neither loupes nor microscopes. In regard to the evacuation of spinal epidural hematomas, 3% (1/33) of attending surgeon respondents nationwide, 17% (2/12) of faculty, 10% (1/10) of pre-survey, and 30% (3/10) of post-survey residents endorsed using neither loupes nor microscopes.

**Figure 4:**
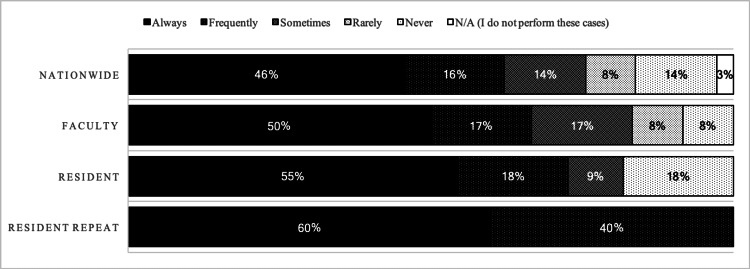
The proportions of neurosurgeons who use loupes with headlights for procedures

During spinal microdiscectomy, 3% (1/29) of attending surgeon respondents nationwide, 8% (1/12) of faculty, 10%(1/10) of pre-survey, and 30% (3/10) of post-survey residents endorsed using neither loupes nor microscopes. For carpal/cubital tunnel surgery, 6% (1/18) nationwide, 13% (1/8) of faculty, 13% (1/8) of pre-survey, and 40% (4/10) of post-survey residents endorsed using neither loupes nor microscopes. Lastly, for clipping of a circle of Willis aneurysm, 0% (0/16) of attending surgeons nationwide, 0% (0/7) of faculty, 10% (1/10) of pre-survey, and 33% (3/9) of post-survey residents endorsed using neither loupes nor microscopes. Figure 4 demonstrates the frequency in which surgeons and residents use the aforementioned intraoperative devices.

## Discussion

After a review of this data, it appears that resident usage patterns had a tendency to align with faculty at their institution (between pre-survey and post-survey). The most frequently used technologies across all evaluated surgical procedures included loupes, which were employed in almost every surgery as well as frequent usage of surgical microscopes and US. All residents and faculty were involved in mentor-mentee monthly meetings, and all were from a single neurosurgery training program. This identifies that the practice parameters of residents closely model those behaviors portrayed by their attending mentors. The attending mentors, on the other hand, did appear to differ from those attending surgeons who were surveyed at academic institutions on a national level.

Apart from peripheral nerve decompression, the use of US by residents is more closely aligned with their mentor faculty than the use at the national level by surgeon attendings. Regarding the use of microscopy, except for aneurysms, subdural hematoma evacuation, and intradural spinal tumor resection, resident practices are more closely aligned with faculty compared to nationwide usage by attending surgeons. The most variation was seen with surgical loupes, with residents using loupes more than both faculty and surgeon attendings at the national level. This may be explained by the initial stages of surgery being performed by residents, prior to the introduction of the microscope into the surgical field.

It appears that the higher frequency of surgical loupes used by residents when compared to faculty and neurosurgeons nationwide further highlights the importance of this equipment in training neurosurgical residents. By learning to use all the necessary equipment early and often, residents’ training is optimized. Furthermore, faculty neurosurgeons using this equipment at a higher rate than neurosurgeons nationwide show the importance of leading by example; as faculty neurosurgeons use this equipment, residents are encouraged to use it as well. This paper also demonstrates the ease of incorporation of nationally recognized learning methods when discussed in an academic session and modeled by the faculty. The resident behavior utilizing US, microscope, loupes, and headlights increased after presenting the data and discussion with the faculty.

Additionally, this analysis identifies that while surgical loupes are frequently an investment on the part of the operating surgeon or resident, they are a crucial technology for an appropriate neurosurgical standard of care. Loupes are not only important for residents working, but for faculty training residents as well. Faculty should be using the intraoperative equipment at rates similar to or greater than the nationally accepted practice to help residents become comfortable using the equipment and receive the best training. There often exist discrepancies between faculty and resident perceptions of intraoperative learning and treatment [3]. Increased usage of common intraoperative equipment may narrow these perceptive discrepancies.

Hospital/healthcare policy surrounding loupes appears to not have adapted to the necessity of loupes for surgical work. While Australia and the United Kingdom offer tax deductions for purchasing loupes, there appears to be no such policy in the United States [4]. At least among dental practitioners, price is the main barrier to usage [5]. There is no reason to believe that this is any different for surgical residents. While costs may be high for purchasing equipment, the return on investment for residents is high. Residents bring substantial economic benefits, particularly with relative value units (RVUs) with up to 8172 work RVUs produced per resident every two years [6]; thus, subsidizing equipment for residents may produce outsized benefits in return with regard to the quality of medical care. Increased financial expenses, especially with student debt, may contribute to increased hardships for residents [7,8]. Therefore, minimizing expenses for residents can disproportionately decrease stress. The use of bifocal safety glasses with magnification offers a low-cost alternative to standard loupes that are used by some institutions by physician assistants [9]. Alternatively, some departments consider loupes an elective cost that could be cut from departmental expenditures on residency programs [10].

Early acquisition of loupes ensures that residents stay on par with national trends in training and in practice; early acquisition of surgical loupes should be encouraged by programs. This may necessitate additional funding for these critical devices to improve access for residents early in their training so that they may practice safely and appropriately. Loupes are a vital tool for residents and attendings, and this survey offers further support for the use of loupes and developing policy to make equipment more accessible for all.

As with most survey-style studies, the limitation of this paper includes reporting bias that arises when asking participants to report their own behaviors. It is also notable that the majority of programs did not respond, making this study susceptible to nonresponse bias. As such, reported usage may overall be over- or under-represented. However, we believe that, overall, practice patterns align with those of their mentors.

## Conclusions

Various intraoperative equipment is necessary for neurosurgical procedures. These include an operative microscope, US, and loupes with a headlight. The necessity of these pieces of equipment makes them vital in the training of residents as well. As such, they should be available to residents from the onset of training to promote the highest quality of learning. Faculty should encourage the use of this equipment by leading by example, and residents, in turn, should use all the available equipment as often as possible to maximize the quality of their training. This study is limited by bias susceptible to survey-style studies, including reporting and nonresponse bias. However, we have identified that the practice patterns of residents closely model those behaviors portrayed by their attending mentors at their institution. The attending mentors, on the other hand, did appear to differ from those attending surgeons who were surveyed at academic institutions on a national level.

## Figures and Tables

**Figure 5:**
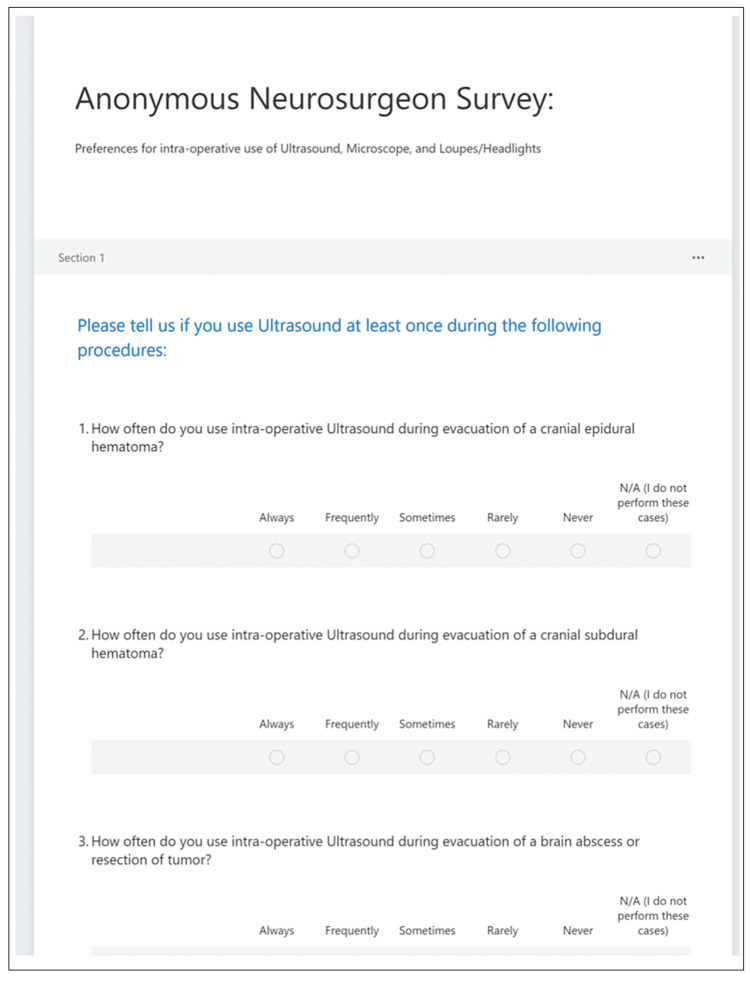
Sample of survey questions sent to 102 Accreditation Council for Graduate Medical Education (ACGME) Neurosurgery programs in the United States as well as core faculty and residents from a single institution
